# Annotations

**Published:** 1889-09-14

**Authors:** 


### Annotations.

**Coaxing Sleep.** Sleeplessness is on the increase, and is likely to extend still further. The more highly developed the brain, the more unstable, probably, is its equilibrium. Every brainworker may convince himself of this by reflecting how sound and unbroken was the sleep of his boyhood compared with the wakefulness and brain activity that now haunt his pillow on the smallest provocation. But of all the ills for which drugs should not be resorted to except in direst extremity, sleeplessness is the chief. Sleeping draughts in the hands of the uninstructed are among the most dangerous and injurious things known. Not only are they edged tools which when played with may cut and kill at the moment, but they are poisons which, when persevered with, often produce a condition of alternate imbecility and anguish infinitely worse than death. The only really safe and justifiable method of treating continued sleeplessness is one which restores the brain to normal daily activity and nightly quiescence. The brain must be taught and coaxed to take its sleep as naturally as a hen takes her roost. But the brain is wilful when mismanaged, and will not be coaxed into sleep at night if it is insanely used in the day. Every use to which the brain can be put is an insane use if it be incompatible with health. Tried by this standard, there are few persons more insane than doctors, unless, indeed, it be men of pure science, and great authors. What a commentary on the practical faith of doctors and great authors is their constant habit of overwork, overstudy, and consequent enfeeblement of both brain and body! There is not the slightest doubt that physiology teaches a judicial, equable, and moderate way of life, just as Christianity teaches self-denial and universal friendliness. But a doctor who practises the physiology he preaches is as rare as a priest who loves all men with the same tender solicitude as he regards himself. Sleep can be coaxed, persuaded, nay, compelled by absolute and prolonged obedience to the physiological gospel. The professor of religion is unhappy because he is only religious with half his heart. In the same way the man who would fain have sleep, good appetite, sound digestion, and health, often fails to secure any of these inestimable blessings for no other reason than that he is unwilling to pay the price. The religion of the body has its ideal as well as the religion of the soul; and he who would rise to the highest altitude must begin by crucifying the less rational desires. It is worth remembering that "the sleep of the labouring-man is sweet, whether he eat little or much; but the abundance of the rich will not suffer him to sleep."

**Toughened Nerves.** We are all beginning to find out—indeed, many of us have found out already—that the "coming man" is the man of tough nerve fibre. Steady, stolid, central sense and unlimited staying power, these are the notes of the present and the future among those who are making the present and the future. Brilliancy, however brilliant, dash, however dashing, and rush, however tremendous and apparently irresistible, cannot overcome the forces of resistance which stand in the way of ambition or great purpose. Sense and staying power are alone equal to the occasion. Natural capacity may be born with a man, sense comes from experience; but how are toughened nerves and staying power to be obtained? The farm labourer inherits tough nerves, but "tough" and "toughened" things are not always the same. What is wanted is the clever man's cleverness without his tendency to break down. How is that to be come at? Fortunate would be the quack who could sell the means of producing it in a thirteenpence-halfpenny pill-box! Famous and rich would be the physician who could concentrate the essence of it in a three-guinea prescription! But it is not to be done. Enduring nerve power is a coy goddess, who is not to be won by a mere sonnet or the gift of a bracelet. And yet without such power the most splendid brains and the noblest purposes may end in the merest futility. Are there any means known to science or plain sense whereby toughened nerves may be secured and retained? Undoubtedly! Patience is on friendly terms with them. The impatient man is constantly quarrelling with his own nerves, as well as with other people's. The patient man avoids friction, which wears away nerves as well as other things. Sound sleep is the intimate companion of steady nerves. Sleeplessness is destruction and death to them. Strenuous work within limited hours is a fine nerve tonic, and tends to produce staying power. In fine, for a long race in life, as well as in athletics, a man must "train." Without training he cannot run. And he must train patiently and properly, not too hard. A "spurter" without staying power never did win a long race, and never will. One play would not have made a Shakespeare any more than one swallow makes a summer. It needed many plays to give the world full and unquestioned assurance of the real nature of the man. So a single year well spent, a degree well taken, a few newspaper articles well written, one or two public works well done, are not enough for the work of a whole lifetime. Staying power is demanded in order that the noblest gifts of intellect and character may put forth their best efforts, and do the best work which the world needs and demands of the capable man.

**Worry, Work, and Sunstroke.** The sudden and altogether unlooked for death of Mr. J. F. B. Firth, Deputy-Chairman of the London County Council, at the early age of forty-seven, furnishes a striking object-lesson to the too strenuous and too persistent struggling fighters of this generation. Mr. Firth was a man who worked and worried. He took things to heart and did them with his whole mental and moral energy. That is how things should be done, no doubt; but when a man fights with all his soul, he should not fight at all times. He should, moreover, acquaint himself with all the conditions of successful and continuous fighting. Mr. Firth died of sunstroke—that is, of heat-apoplexy. It is stated that the heart was also ruptured. Excessive work and worry bring about conditions that are highly dangerous if the head be exposed to the sun's rays. Work and worry of brain have the effect of filling the brain too full of blood, of weakening the walls of the cerebral bloodvessels, and so causing them to dilate, of exhausting the nerve fibres which give to the walls of the bloodvessels their contractile power, and so inducing a state of permanent brain congestion. The heat of the sun acts as a congester of the brain; but if the sun finds a congested condition ready to his hands, so to speak, a condition that long-continued habits of overwork and worry have made almost habitual and permanent, a very little additional heat is all that is needed to produce sunstroke. It is a most serious misfortune that the average man has no idea whatever how nicely balanced are the conditions which make for safety on the one hand against those that make for apoplexy and death on the other. If Mr. Firth had not been suffering from overstrain, the degree of heat which killed him might have done him no more harm than is implied in a slight headache. If he had had a holiday long enough to have diminished the cerebral congestion, and to have restored the energy of those vasometer nerves which secure the adequate contraction of the bloodvessels, he might have been still alive, and have continued to live until he was almost twice the age at which he died. Surely, it is more than a pity that even able and cultured men do not think it worth while to acquire so much as an elementary knowledge of those conditions of brain and health which conduce to continued efficiency and safety. If a man walks over a precipice with his eyes open, we call him a suicide; but a man may go through whole regions of life with his eyes shut when they might be open, and we hold up our hands in amazement if he now and again tumbles into a pit which would have been plain and conspicuous before him had he but taken the trouble to look. Will half-a-dozen persons in all England understand the lesson which Mr. Firth's sudden and untimely death teaches them? Probably not. The strenuous man should work interruptedly, and the interruptions should be of sufficient duration to bring the brain back to its natural state of quiet and repose. When he is in the midst of his work he should avoid exposure to the extreme heat of the sun, in any country, as he would avoid a mad dog if he saw one on the road, or a savage bull if he met him in the field.

